# Magnetic Resonance Imaging Findings After Superior Capsule Reconstruction

**DOI:** 10.1016/j.asmr.2020.09.008

**Published:** 2021-01-30

**Authors:** Dale Nicholas Reed, James Tyler Frix, James Mitchell Frix

**Affiliations:** a Associates in Orthopedics and Sports Medicine, Dalton, Georgia, U.S.A.; b AU/UGA Medical Partnership, Athens, Georgia, U.S.A.

## Abstract

**Purpose:**

To evaluate graft properties on magnetic resonance imaging (MRI) after superior capsular reconstruction (SCR) to help improve our understanding of postoperative imaging.

**Methods:**

We identified consecutive patients who underwent SCR by a single surgeon and who had postoperative MRIs available. MRIs were analyzed to look for common postoperative findings on imaging.

**Results:**

Ten consecutive patients with an average age of 58 years who underwent SCR by a single surgeon had postoperative MRIs on average 404 days from surgery. Eight patients had completely intact grafts on follow-up MRI. All intact grafts were similar with homogenous appearance on all coronal images. All patients displayed some trace fluid with mild heterogenous signal at the level of the glenoid, which could represent failure of the graft to completely incorporate at the level of the glenoid or could be normal in the postoperative setting since all eight intact grafts displayed this finding. None of the patients with intact grafts had bony edema noted on either the glenoid or humeral side. Four of 8 patients were noted to have trace bony edema at the level of the lateral acromion. One patient had complete disruption on the glenoid side. One patient had partially intact graft that revealed heterogenous appearance of graft.

**Conclusions:**

An intact graft displays a more homogenous signal on consecutive postoperative MRI coronal images than disrupted grafts or partially intact grafts. This suggests that intact grafts have better clinical outcomes than a partially disrupted or completely disrupted graft. However, the finding of heterogenous signal/fluid at the glenoid graft interface in all intact grafts could not be explained in this study.

**Level of Evidence:**

Level IV, therapeutic case series.

Superior capsular reconstruction (SCR) has gained popularity in the treatment of patients with massive and irreparable rotator cuff tears. SCR originally was described using fascia lata autograft and has shown promising results clinically.^1^^,^^2^ Dermal allograft was then proposed as an alternative to fascia lata autograft to prevent graft harvest and save operative time.^3^ Pennington et al.^4^ have shown success using the dermal allograft in decreasing pain and improving function in 86 patients. Denard et al.^5^ described success in 70% of 59 patients using dermal allograft. Burkhart et al.^6^^,^^7^ published encouraging results with 90% reversal of pseudoparalysis in 10 patients with SCR. Burkhart recently published clinical outcomes at 2 years postoperatively and demonstrated 85% success rate with durable clinical outcomes.^7^ Several systematic reviews have shown the SCR to be viable option in decreasing pain and improving function with minimal complications and reoperation rates in the short term.^8^^,^^9^

Even though there has been an explosion in literature about SCR, little is known about findings on the postoperative magnetic resonance imaging (MRI). In the primary papers on dermal allograft for SCR, only 34 MRI scans were obtained postoperatively.^4^, ^5^, ^6^ Of these 34 MRI scans, very little is mentioned except graft intact, partially intact, or completely disrupted. This can make reviewing postoperative MRIs challenging for the performing surgeon. We felt that any additional information gained from reviewing postoperative MRIs on consecutive patients, regardless of how they were doing clinically, could strengthen our understanding of postoperative imaging after SCR.

The purpose of this study was to evaluate graft properties on MRI after SCR to help improve our understanding of postoperative imaging. Our hypothesis was that postoperative MRIs of intact SCR grafts would show homogeneous signal on 3 consecutive coronal images.

## Methods

The study included consecutive patients who underwent arthroscopic SCR by a single surgeon using dermal allograft (3-mm nominal thickness, ArthroFLEX 301; Arthrex, Naples, FL). Inclusion criteria were patients who had full-thickness rotator cuff tear (supraspinatus and infraspinatus tendons) who were found to be irreparable at the time of surgery. Exclusion criteria were patients with full-thickness retracted subscapularis tears, full-thickness cartilage defects, patients who were smokers, had uncontrolled diabetes defined as A1C >8, or who were unable to comply with postoperative protocol for any reason.

The surgical technique for the SCR has been described by multiple authors.^3^^,^^10^ Our preference is the beach-chair position. Diagnostic arthroscopy is performed, and concomitant pathology is addressed. Partial upper border subscapularis tears that are noted are repaired using the single-row technique with 1 to 2 anchors. The biceps tendon is tenotomized if still present. The superior glenoid is prepared to get a bleeding, bony bed, and 3 anchors are placed, if possible, in an equally spaced pattern. Secondary to size of glenoid, some patients only had 2 glenoid anchors placed. Typically, 4 anchors are placed on humeral side to secure the graft. Two anchors are placed medial on the footprint to secure the graft and 2 anchors are placed laterally for a suture bridge configuration. Any remaining posterior rotator cuff tendon is repaired side to side with the graft. We always repair upper border of subscapularis to anterior edge of graft as well.

All included patients had preoperative and postoperative MRI on affected shoulder. The pre- and postoperative MRIs were analyzed to look for any changes that occurred after SCR was performed. Preoperative MRI evaluation included number of tendons torn, size of tear in both the coronal and sagittal planes, and Goutallier score. Subscapularis integrity was noted at the time of surgery as well as the number of arthritic changes that was not seen on preoperative imaging. All patients had both preoperative and postoperative MRIs performed. Graft integrity was defined as completely intact (no disruption from glenoid to humerus on any coronal images T1 and T2), partially intact (at least 1 coronal image with graft bridging from glenoid to humerus), and completely disrupted (no coronal images showing graft bridging from glenoid to humerus). Postoperative MRI was used to evaluate the integrity of the graft on multiple consecutive coronal images, as described by Burkhart et al.^6^ Postoperative MRIs also were used to analyze the glenoid–graft interface, the homogeneity of the graft, and the humoral–graft interface. Any fluid about the shoulder and any bony changes were noted as well. The integrity of the subscapularis repair was evaluated as well as the Goutallier score.

Postoperatively, all patients were placed in sling immobilization with abduction pillow for 8 to 10 weeks. No active range of motion of above chest level until after 3 months. No strengthening or lifting until after 6 months postoperatively.

## Results

Ten consecutive patients were included in the study. Seven patients were male and 3 were female. The average age was 58 years. Three patients were involved in workers compensation claims. No patients were smokers or used any type of tobacco products. No patients had more than 1 to 2 drinks per week. Only 1 patient had had previous rotator cuff surgery. Interestingly, 7 of 10 patients had subscapularis repair done at the time of surgery. Only 2 patients were found to have grade 1 to 2 changes on the humeral head at the time of surgery. No patients experienced any postoperative complications.

The average time from preoperative MRI to surgery was 127 days. The average time from surgery to postoperative MRI was 404 days. Nine of 10 patients had preoperative tangent sign. The average preoperative Goutallier score for the supraspinatus was 3.3, infraspinatus was 2.5, and subscapularis was 0.6. The average tendon retraction in the coronal plane was 4.2 cm. The average tendon tear size in the sagittal plane was 4.07 cm (Table 1).

**Table 1:** Preoperative MRI Findings

Sex	Age, y	Previous Surgery	Goutallier Score			Arthritis	Subscapular Tear	Retraction, cm	
			Supra	Infra	Sub			Coronal	Sagittal
Male	52	No	3	2	0	None	No	4.3	4
Male	53	No	3	1	1	None	Yes, repaired	3.5	4.5
Male	75	No	4	4	1	None	Yes, repaired	4.5	4.2
Male	63	prior RTC	3	2	2	None	Yes, repaired	4.4	4.4
Male	64	No	4	3	1	None	Yes, repaired	6	5.3
Female	46	No	2	2	0	Grade 1 degenerative changes	No	3.3	2.1
Male	54	No	3	3	1	None	Yes, repaired	4.5	5.2
Female	54	No	4	2	0	Grade 2 changes on humeral head	Yes, repaired	3.7	4
Female	65	No	3	2	0	None	No	3.8	3.4
Male	55	No	4	4	0	None	Yes, repaired	4.1	3.6

NOTE. Shown are the preoperative MRI findings of the 10 patients in this study. Highlighted findings include sex, age, previous surgical intervention, Goutallier scores, glenohumeral arthritis, involvement of the subscapularis tendon, and retraction distance of the supraspinatus tear.

Infra, infraspinatus; MRI, magnetic resonance imaging; Sub, subscapularis; Supra, supraspinatus; RTC, rotator cuff repair.

The average number of anchors used on the glenoid side was 2.6. The average number of anchors used on the humeral side was 4.1. The average postoperative Goutallier score for the supraspinatus was 3.8, infraspinatus was 3.2, and subscapularis was 0.5. Eight patients had completely intact grafts, 1 patient had a partially intact graft, and 1 patient had a completely disrupted graft (Table 2).

**Table 2:** Postoperative MRI Findings

Sex	Age, y	Goutallier Score			Anchors Used		Graft Status	Fluid		Bony Edema
		Supra	Infra	Sub	Glenoid	Humeral		Glenoid Attachment	Humeral Attachment	Acromion
Male	52	4	3	0	3	4	Complete disruption	Graft failure	Graft failure	Graft failure
Male	53	3	1	1	3	4	Completely intact	Trace	Trace	None
Male	75	4	4	1	2	4	Completely intact	Trace	None	None
Male	63	4	4	1	3	4	Completely intact	Trace	None	Present
Male	64	4	2	1	2	5	Completely intact	Trace	None	None
Female	46	3	2	0	3	4	Completely intact	Trace	None	None
Male	54	4	4	1	2	4	Partially intact	Trace	None	None
Female	54	4	4	0	3	4	Completely intact	Trace	None	Trace
Female	65	4	4	0	2	4	Completely intact	Trace	None	Trace
Male	55	4	4	0	3	4	Completely intact	Trace	None	Trace

NOTE. Shown are the postoperative MRI findings of the 10 patients in this study. Highlighted findings include sex, age, Goutallier score, number of anchors used during SCR reconstruction, and SCR graft status at postoperative MRI, including any findings of fluid or bony edema.

Infra, infraspinatus; MRI, magnetic resonance imaging; SCR, superior capsular reconstruction; Sub, subscapularis; Supra, supraspinatus.

**Fig 1:**
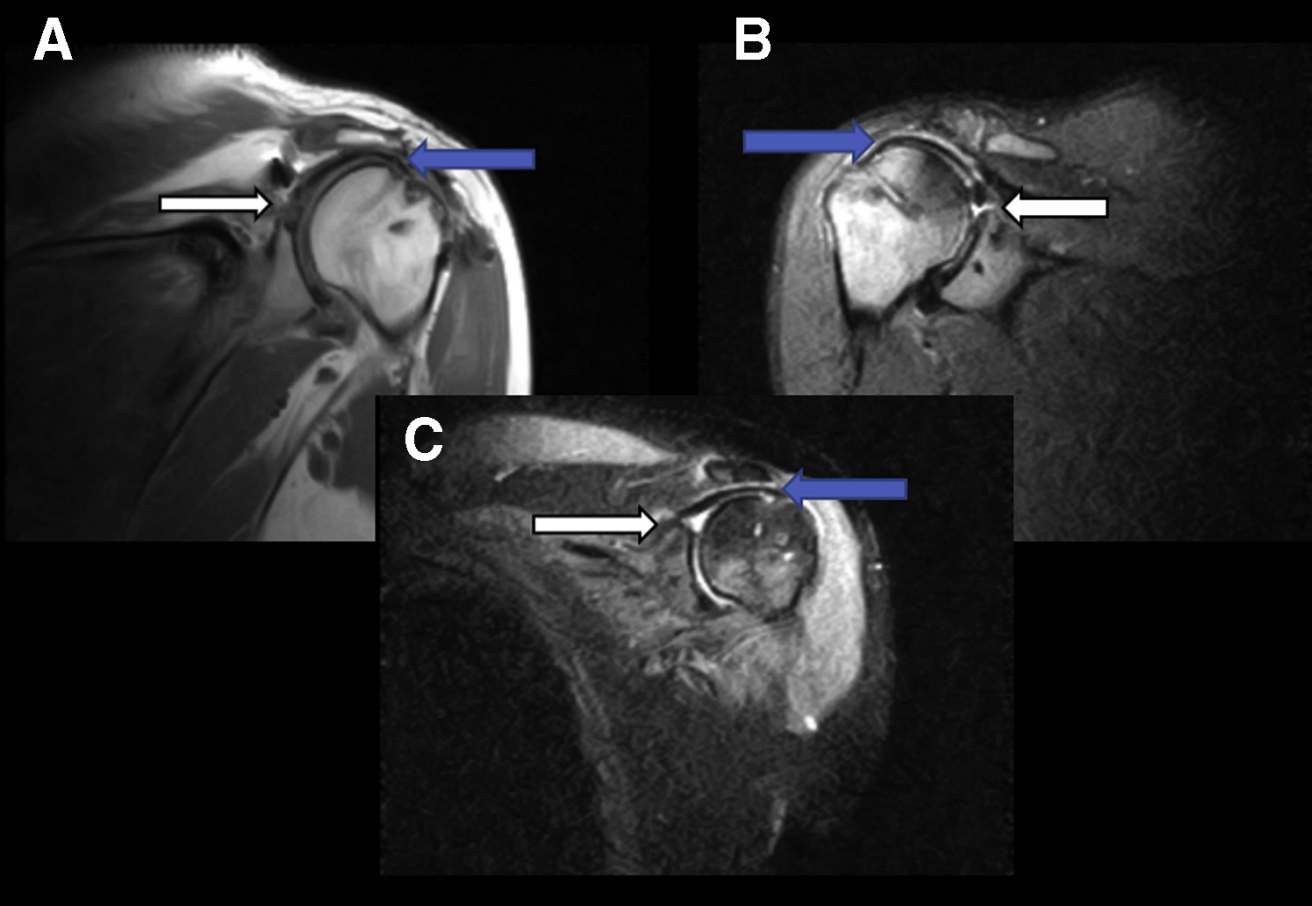
Three completely intact grafts (A, B, and C). These images represent intact grafts on postoperative MRIs after superior capsular reconstruction. The blue arrows denote intact homogeneous graft that can be seen spanning from glenoid to humeral head. White arrows denote heterogenous signal at glenoid, which could represent a normal finding postoperatively or incomplete incorporation at the glenoid graft interface. (A) T1 coronal of left shoulder. (B) T2 inversion recovery coronal of left shoulder. (C) T2 inversion recovery coronal of right shoulder. (MRI, magnetic resonance imaging.)

**Fig 2:**
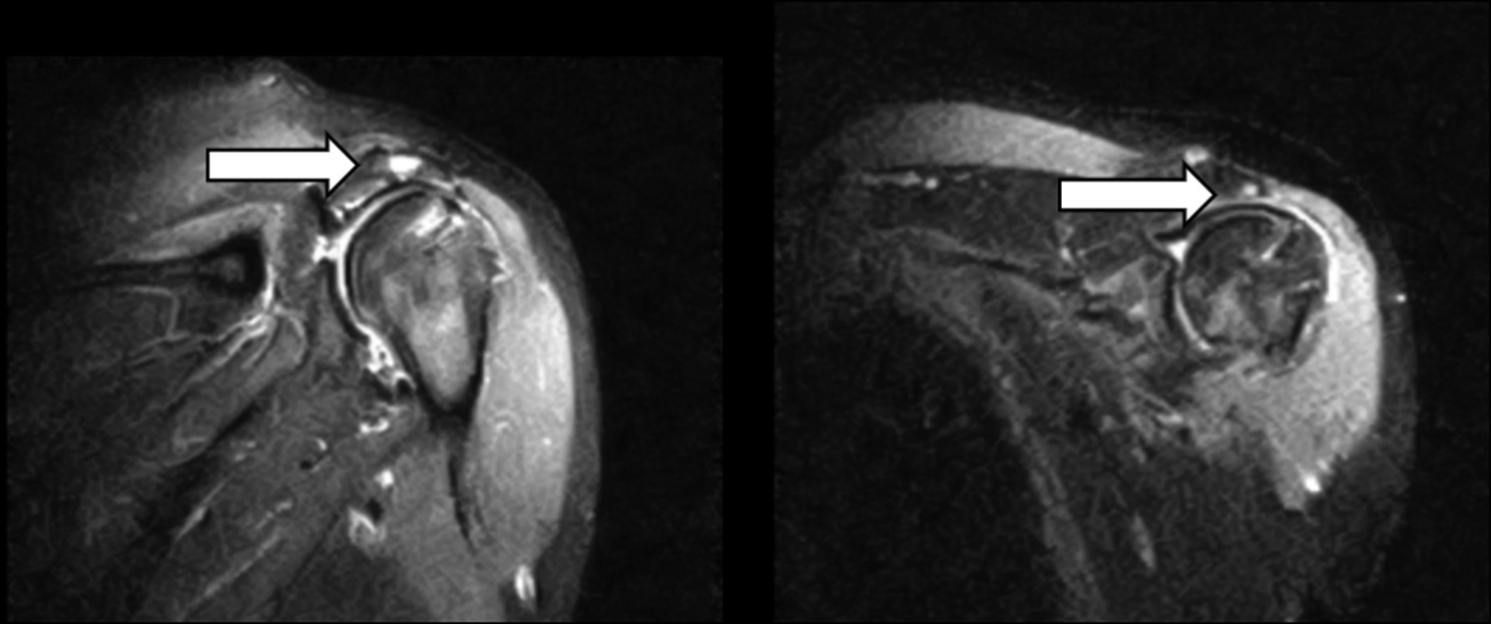
Images depicting bony edema. These images display postoperative MRI bony changes noted after superior capsular reconstruction. The arrows note bony edema at the level of the acromion after SCR. No SAD/acromioplasty was performed at time of procedure. This is likely secondary to continued abnormal contact between the humeral head and acromion. Both figures are T2 inversion recovery coronal images. (MRI, magnetic resonance imaging; SAD, subacromial decompression; SCR, superior capsular reconstruction.)

As noted previously, 8 patients had completely intact grafts on follow-up MRI. All intact grafts looked similar with homogenous appearance within the graft body on all coronal images. All patients displayed some trace fluid with heterogenous signal at the level of the glenoid (Fig 1). This fluid could represent failure of the graft to completely incorporate at the level of the glenoid or could be normal in the postoperative setting, since all 8 intact grafts displayed this finding. Six of 8 patients had a mild amount of fluid noted in joint. Three of 8 had a trace amount of fluid noted in subacromial space. None of the patients with intact grafts had bony edema noted on either the glenoid or humeral side. Four of 8 patients were noted to have trace bony edema at the level of the lateral acromion (Fig 2).

**Fig 3:**
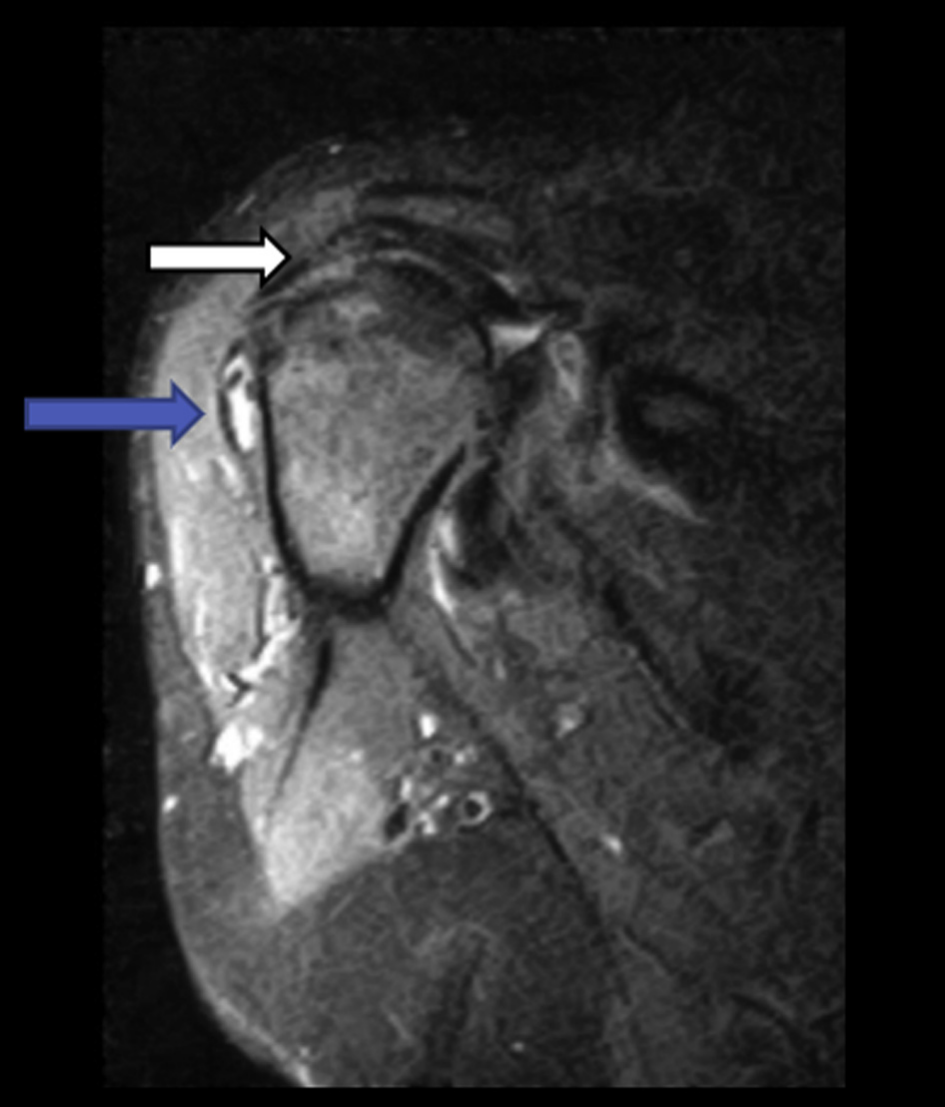
T2 inversion recovery coronal image of partially intact graft. The image displays a postoperative MRI of a patient after superior capsular reconstruction that is partially intact. Note the heterogenous pattern of the graft at white arrow. This finding likely represents graft breakdown, resorption, or only partial vascularization of the graft. Note the increased fluid about the graft in the subacromial space at blue arrow. (MRI, magnetic resonance imaging.)

The patient with the partially intact graft also had subscapularis repair at the time of the procedure. Interestingly, the subscapularis failed as well as the anterior half of the graft. Also, the MRI revealed a heterogenous signal within the body of the graft on coronal images (Fig 3). This was the only graft to have heterogenous signal in the study. This could possibly be secondary to the disruption of the graft and either graft breakdown or only partial vascularization of the graft. Trace fluid was noted around glenoid attachment of graft which was common in all patients. This was the only graft to have more than trace fluid noted about the graft.

**Fig 4:**
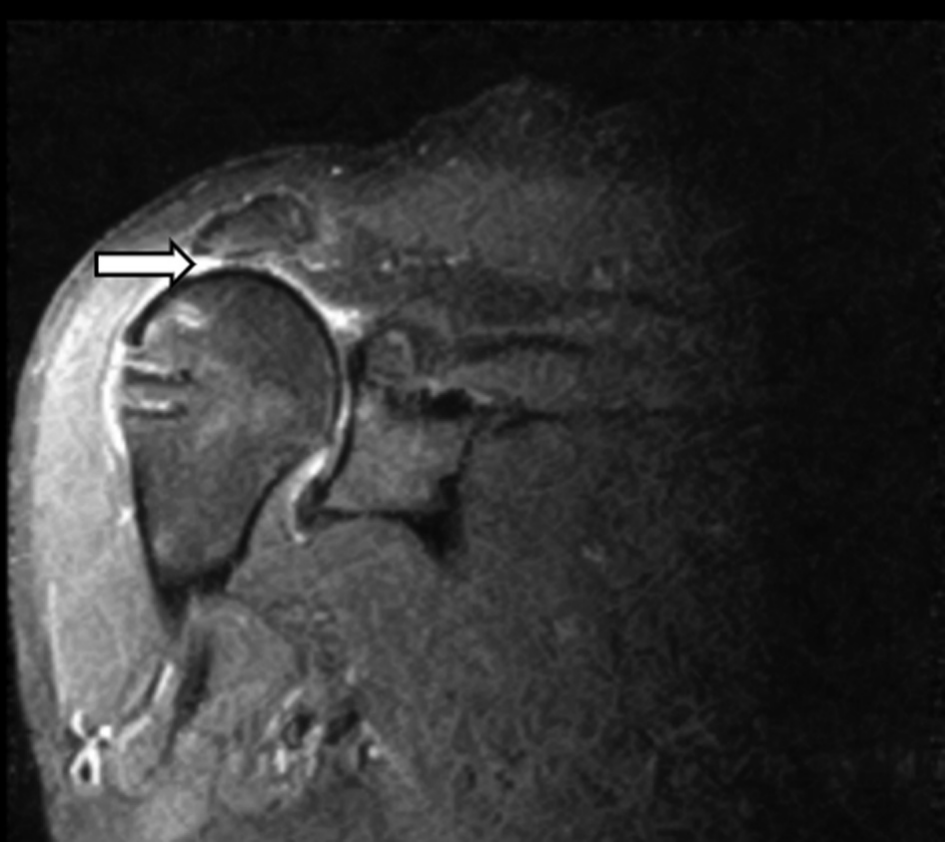
T2 inversion recovery coronal MRI image of the right shoulder. The image displays a postoperative MRI of a patient after superior capsular reconstruction with complete failure. The arrow denotes absence of graft between humerus and acromion. There is no attachment noted to the glenoid on the image which confirms a completely disrupted graft. Note humerus is nearly articulating with undersurface of acromion. (MRI, magnetic resonance imaging.)

**Fig 5:**
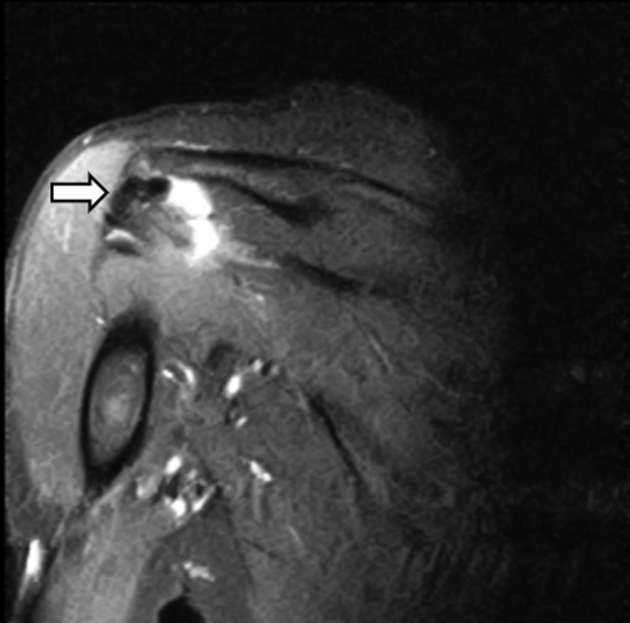
T2 inversion recovery coronal MRI of same shoulder from Figure 1. The image displays a postoperative MRI of a patient after superior capsular reconstruction with complete failure. The arrow denotes the dermal allograft still has some attachment to humerus and has balled up in posterior aspect of shoulder. There is no attachment to the glenoid on any coronal views thus demonstrating complete failure from the glenoid side. (MRI, magnetic resonance imaging.)

The patient with complete disruption of the graft occurred within the first 6 months. The graft failed on the glenoid side (Figs 4 and 5). The patient was revised to a reverse total shoulder arthroplasty and has done well. At the time of surgery, the graft was noted to be incorporated on the humeral side and all anchors were still intact on glenoid side. The failure was at the suture–graft interface.

All patients with intact grafts demonstrated a homogenous signal at humeral attachment with no evidence of fluid at the graft–bone interface between the medial and lateral rows. This likely represents a quiet interface demonstrating a stable humeral attachment. All intact grafts displayed minimal fluid in the joint or in subacromial space. The partially disrupted graft revealed increased fluid in subacromial space compared to intact grafts. It also demonstrated bony edema on the humerus and glenoid, an MRI finding only identified in the single, partially-intact graft. This may represent abnormal contact forces secondary to failure or abnormal strain on the remaining graft that is stressing the graft-bone interface. There were 3 patients with intact grafts who displayed edema on the undersurface of the lateral acromion. This is likely secondary to continued abnormal contact between the humeral head and acromion. No acromioplasty was performed in any of the patients in the study. The Goutallier scores increased in respect to the supraspinatus and infraspinatus and remained stable for the subscapularis. Therefore, even with an intact graft, muscle atrophy of the torn tendons continued.

## Discussion

The findings from our study demonstrate that intact grafts display a more homogenous signal on consecutive coronal images than disrupted grafts or partially intact grafts. The fact that there is some signal noted at the glenoid graft interface could be a normal finding or could represent incomplete incorporation of the graft. However, in our study all patients with an intact graft were happy with their results to date and have had no revision surgery. The graft that was partially intact displayed a more heterogenous signal throughout the body of the graft and likely represents graft breakdown, resorption of the graft, or incomplete vascularization of the graft. The patient with the partially intact graft had not been revised at the time of publication but was not as happy with his outcome when compared to our other patients with intact grafts. The patient with graft failure on MRI was unhappy with result and thus revised to a reverse total shoulder arthroplasty.

Our study adds 10 additional MRI studies to the current literature to give a total of 44 postoperative MRIs in the current literature at time of publication. Burkhart and Hartzler^6^ performed postoperative MRIs on 10 patients and noted 7 completely intact grafts and 3 partially intact grafts with no failures. There was no other mention of additional findings other than intact, partially intact, or disrupted on postoperative MRIs. Their findings were similar to our 10 in the fact that all the patients in their study and ours with intact or partially intact grafts were clinically improved and have not been revised.^6^ Denard et al.^5^ performed MRI on 20 of 67 patients. Of the 20 patients in the study who had postoperative MRI, 11 of 20 had graft failure. The patients were chosen based on those “who were willing to undergo MRI.” They noted which side the graft failed: 7 on humeral side, 3 intrasubstance and 1 on glenoid side. Clinically, patients with intact graft did better than those with graft disruption. They made no other mention of postoperative MRI findings.^5^ Our current study complements the finding that patients with intact graft do better than those with graft disruption. Pennington et al.^4^ had postoperative MRIs on 4 of 88 patients and only in patients who were not doing well. They made no mention of MRI findings other than noting failure.^4^ Hirahara et al.^11^ included 9 SCRs with follow-up ultrasound but no MRI.

Based on our observations and the aforementioned studies, an intact graft appears to have better clinical outcomes than a partially disrupted or completely disrupted graft. Our imaging data show intact graft demonstrate a more homogenous appearance on both T1 and T2 coronal imaging with the partially intact graft demonstrating a more heterogenous appearance. Also, all intact grafts in our study displayed a trace amount of fluid with heterogenous signal at the glenoid attachment. This finding needs further research to be certain that this is a normal finding with intact grafts or a sign of incomplete incorporation at the level of the glenoid.

This study adds to the existing literature regarding SCR and provide a framework for interpretation of postoperative MRIs. It is our hope that this paper will prompt other surgeons currently performing SCR to review their patients’ MRI findings to help strengthen the current understanding of MRI findings and their correlation with clinical outcomes.

### Limitations

Limitations of this study are noted with sample size being an obvious weakness; however, providing free MRIs with radiologist reading is difficult in the clinical setting. Another limitation is the lack of correlation of imaging findings to clinical outcome scores. An additional limitation is that only one of our patients was found to have a partially intact graft on postoperative MRI.

## Conclusions

An intact graft displays a more homogenous signal on consecutive postoperative MRI coronal images than disrupted grafts or partially intact grafts. This suggests that intact grafts have better clinical outcomes than a partially disrupted or completely disrupted graft. However, the finding of heterogenous signal/fluid at the glenoid graft interface in all intact grafts could not be explained in this study.

## Supplementary Data

ICMJE author disclosure forms

## References

[1] Mihata T., Lee T.Q., Watanabe C. (2013). Clinical results of arthroscopic superior capsule reconstruction for irreparable rotator cuff tears. Arthroscopy.

[2] Mihata T., Watanabe C., Fukunishi K., Ohue M., Tsujimura T., Kinoshita M. (2011). Arthroscopic superior capsular reconstruction restores shoulder stability and function in patients with irreparable rotator cuff tears: A prospective study (SS-15). Arthroscopy.

[3] Hirahara A.M., Adams C.R. (2015). Arthroscopic superior capsular reconstruction for treatment of massive irreparable rotator cuff tears. Arthrosc Tech.

[4] Pennington W.T., Ba Bartz, Pauli J.M., Walker C.E., Schmidt W. (2018). Arthroscopic superior capsular reconstruction with acellular dermal allograft for the treatment of massive irreparable rotator cuff tears: Short-term clinical outcomes and the radiographic parameter of superior capsular distance. Arthroscopy.

[5] Denard P.J., Brady P.C., Adams C.R., Tokish J.M., Burkhard S.S. (2018). Preliminary results of arthroscopic superior capsule reconstruction with dermal allograft. Arthroscopy.

[6] Burkhart S.S., Hartzler R.U. (2019). Superior capsular reconstruction reverses profound pseudoparalysis in patients with irreparable rotator cuff tears and minimal or no glenohumeral arthritis. Arthroscopy.

[7] Burkhart S.S., Pranckun J.J., Hartzler R.U. (2020). Superior capsular reconstruction for the operatively irreparable rotator cuff tear: Clinical outcomes are maintained 2 years after surgery. Arthroscopy.

[8] Catapano M., de Sa D., Ekhtiari S., Lin A., Bedi A., Lesniak B.P. (2019). Arthroscopic superior capsular reconstruction for massive, irreparable rotator cuff tears: A systematic review of modern literature. Arthroscopy.

[9] Sochacki K.R., McCulloch P.C., Lintner D.M., Harris J.D. (2019). Superior capsular reconstruction for massive rotator cuff tear leads to significant improvement in range of motion and clinical outcomes: A systematic review. Arthroscopy.

[10] Burkhart S.S., Denard P.J., Adams C.R., Brady P.C., Hartzler R.U. (2016). Arthroscopic superior capsular reconstruction for massive irreparable rotator cuff repair. Arthrosc Tech.

[11] Hirahara A.M., Andersen W.J., Panero A.J. (2017). Superior capsular reconstruction: Clinical outcomes after minimum 2-year follow-up. Am J Orthop (Belle Mead NJ).

